# Highlight: Forging Cheaters in Iron-Limited Microbial Communities

**DOI:** 10.1093/molbev/msae072

**Published:** 2024-04-25

**Authors:** Casey McGrath

Competition and cooperation are fundamental forces that govern the evolutionary and ecological dynamics among species. The balance between these forces varies across ecological contexts, with some environments favoring cooperative behaviors that promote mutual benefit, while others reward competitive strategies that maximize individual fitness. Among microbial communities, chemicals that are secreted into the environment provide opportunities for both cooperation and exploitation, giving rise in some cases to microbial “cheaters.” These cheaters exploit the cooperative behaviors of their counterparts, benefitting from the secreted compounds without paying the metabolic costs of production. In a new article published in *Molecular Biology and Evolution*, researchers from the University of Wisconsin-Madison and Vanderbilt University reveal the evolutionary history of secreted iron uptake molecules in yeasts, shedding new light on the cooperative and competitive dynamics that shape iron-limited microbial communities (Sun et al. 2024).

Most organisms require iron for numerous biological processes but are unable to absorb the most common form of iron in the environment. Iron is, therefore, often a limited resource in biological communities. To overcome this scarcity, microorganisms have evolved the ability to scavenge iron from the environment using siderophores, molecules with a high affinity for the type of iron found in the environment. Siderophores are synthesized inside the cell and then secreted into the environment, where they bind to iron; the iron-bound molecules must then be imported back into the cell before the iron can be released and used in cellular metabolism. Siderophores secreted into the environment can be exploited by cheaters who gain a fitness advantage from taking up iron-bound siderophores without investing energy in their production.

While most yeasts are unable to produce siderophores, a research team led by Chris Hittinger found that yeasts in the *Wickerhamiella*/*Starmerella* (W/S) clade could produce a siderophore called enterobactin. The genes required to synthesize enterobactin were apparently horizontally transferred from an ancient bacterium into the ancestor of W/S yeasts (Kominek et al. 2019). Intriguingly, however, the W/S yeasts had no apparent way to reimport the enterobactin siderophore once it was bound to iron. “We did not find any bacterial gene coding for an enterobactin transporter in their genomes,” says Liang Sun, lead author of the new paper. “Secreting enterobactin without bringing it back into the cell for iron uptake would not be a smart move for a yeast cell, so we were very curious as to how those yeasts could potentially utilize the iron bound to enterobactin.”

To solve this puzzle, the team searched the genome of *Starmerella bombicola* for an alternative mechanism for siderophore transport. Through targeted gene disruption experiments and phylogenomic analyses, the team identified a gene known as *ENB1* as crucial for the uptake of enterobactin-bound iron in *S. bombicola*. Surprisingly, *ENB1* is an ancient fungal gene that is likely to date back hundreds of millions of years, predating the divergence of the fungal lineages Basidiomycota and Ascomycota.

Further analyses revealed a complex history of *ENB1* within yeasts. The researchers proposed that *ENB1* was horizontally transferred from an ancestor of the W/S clade to an ancient lineage of Saccharomycetales, the group that includes *Saccharomyces cerevisiae*, which is used to make bread, beer, and wine. This transfer, along with subsequent gene duplications and losses, has shaped the patchy distribution of enterobactin utilization currently observed among yeasts.

These findings have several interesting implications for the history of iron uptake in yeast. As enterobactin uptake apparently predates the ability to produce enterobactin in W/S yeasts, the ancestors of this clade were likely cheaters who benefited from the production of enterobactin by other microbes in their environment. Subsequently, the W/S clade acquired enterobactin biosynthesis genes from a bacterium within an ecological context in which being a producer was more advantageous than being a cheater.

Based on what is known about the distribution of these yeasts, the authors of the study propose that this occurred in an insect gut, where competition for iron among bacteria, yeasts, and the host can be fierce. The ability of W/S yeasts to produce enterobactin and to import it using the Enb1 transporter may have provided a fitness advantage in this highly competitive, iron-limited environment. In contrast, the retention of *ENB1* in cheaters like *S. cerevisiae* “may be associated with ecological niches where bacterial and fungal cohabitants produce enterobactin in response to iron scarcity,” according to the study's authors. “Conversely, the loss of *ENB1* may have occurred in yeasts dwelling in environments with relatively high iron availability or where enterobactin producers are absent.”

While these results are intriguing, additional research is needed to fully uncover the mechanisms by which the fungal and bacterial enterobactin genes became integrated in W/S yeasts. According to Sun, these genes must be tightly coregulated, as “unbalanced secretion and import of enterobactin could hinder iron uptake and subsequently lead to growth defects in the yeasts.” Unfortunately, Sun notes that the metabolic and regulatory networks of these yeasts are not well understood, which could make future studies challenging: “Studying the regulation of this particular pathway may therefore require additional effort to fill in some of these gaps.” Despite these hurdles, this system offers a unique model for further research into the evolutionary dynamics of siderophore transporters in yeasts and their role in promoting cooperation and cheating within microbial communities.

***Want to Learn More?*** Check out these other recent articles from *Molecular Biology and Evolution* highlighting the role of cooperation and competition in shaping microbial evolution.

1. New Evolutionary Insights into RpoA: A Novel Quorum Sensing Reprograming Factor in *Pseudomonas aeruginosa* (Cai et al. 2023)
2. The Architecture of Metabolic Networks Constrains the Evolution of Microbial Resource Hierarchies (Takano et al. 2023)
3. Large-Scale Identification of Known and Novel RRNPP Quorum-Sensing Systems by RRNPP_Detector Captures Novel Features of Bacterial, Plasmidic, and Viral Coevolution (Bernard et al. 2023)
